# Neural Mechanisms Underlying Time Perception and Reward Anticipation

**DOI:** 10.3389/fnhum.2018.00115

**Published:** 2018-03-21

**Authors:** Nihal Apaydın, Sertaç Üstün, Emre H. Kale, İpek Çelikağ, Halise D. Özgüven, Bora Baskak, Metehan Çiçek

**Affiliations:** ^1^Department of Anatomy, School of Medicine, Ankara University, Ankara, Turkey; ^2^Brain Research Center, Ankara University, Ankara, Turkey; ^3^Department of Physiology, School of Medicine, Ankara University, Ankara, Turkey; ^4^Department of Psychiatry, School of Medicine, Ankara University, Ankara, Turkey

**Keywords:** time perception, reward anticipation, fMRI, dopaminergic pathways

## Abstract

Findings suggest that the physiological mechanisms involved in the reward anticipation and time perception partially overlap. But the systematic investigation of a potential interaction between time and reward systems using neuroimaging is lacking. Eighteen healthy volunteers (all right-handed) participated in an event-related functional magnetic resonance imaging (fMRI) experiment that employs a visual paradigm that consists monetary reward to assess whether the functional neural representations of time perception and reward prospection are shared or distinct. Subjects performed a time perception task in which observers had to extrapolate the velocity of an occluded moving object in “reward” vs. “no-reward” sessions during fMRI scanning. There were also “control condition” trials in which participants judged about the color tone change of the stimuli. Time perception showed a fronto-parietal (more extensive in the right) cingulate and peristriate cortical as well as cerebellar activity. On the other hand, reward anticipation activated anterior insular cortex, nucleus accumbens, caudate nucleus, thalamus, cerebellum, postcentral gyrus, and peristriate cortex. Interaction between the time perception and the reward prospect showed dorsolateral, orbitofrontal, medial prefrontal and caudate nucleus activity. Our findings suggest that a prefrontal-striatal circuit might integrate reward and timing systems of the brain.

## Introduction

Perception of time and thus coordination of temporal sequences of events in our internal and external environment is vital to adapt to the world around us. Although time is a fundamental dimension of life, the neural mechanisms underlying time perception are still unclear (Ivry and Spencer, 2004a,b; Lewis and Walsh, 2005; Burr and Morrone, 2006). Is the temporal processing in brain dependent on a specialized system or is it represented by specialized neural networks, or is it regionally perceived depending on the task? This is one of the most fundamental questions which has not been yet properly answered to fully elucidate how the human brain perceives time. The metrical representation of time was generally explored under two categories; duration estimation (explicit timing) and temporal expectation (implicit timing). Basal ganglia, supplementary motor area (SMA), cerebellum, and prefrontal cortex (Coull and Nobre, 2008; Coull et al., 2011) as well as inferior parietal and insular cortex (Ferrandez et al., 2003; Pouthas et al., 2005; Üstün et al., 2017) have been suggested as responsible for explicit timing.

Among the brain regions which were suggested as to have a functionally discrete role in time perception; the dorsal striatum of the basal ganglia and, more specifically, its ascending nigrostriatal dopaminergic pathway suggested to be the most crucial as shown by converging functional neuroimaging, neuropsychological, and psychopharmacological investigations in humans, as well as lesion and pharmacological studies in animals (Coull et al., 2011). Studies using monkey experiments showed that the dopaminergic neurons fire depending on the occurrence and the timing of the rewards (Hollerman and Schultz, 1998; Waelti et al., 2001). In a study of experimental striatum lesion in rat, it was observed that lesioned rats pushed the pedal later than the normal rats to receive a reward (Matell et al., 2003). In addition, dopaminergic agents have systematically deteriorated the performance of timing responses. Administration of dopamine agonists leads to a reduction in perceived time, while antagonists lead to prolongation (Ivry and Spencer, 2004b).

Reward and punishment are also known to be effective upon our future decisions and indeed reward has been shown to promote human performance in multiple task domains. It was suggested that the key brain regions for reward system are the nucleus accumbens (NAc) and the ventral tegmental area (VTA) (Rolls, 2000; Schultz, 2000; Schultz et al., 2000; Kelley and Berridge, 2002; Wise, 2002; Stefani and Moghaddam, 2006; Hikosaka et al., 2008). Recent studies have shown that the striatal and midbrain areas including the entire ventral striatum (VS) and the dopamine neurons of the substantia nigra (SN), are also involved in the reward circuit (Haber and Knutson, 2010). The VS was shown to connect to orbitofrontal cortex and anterior cingulate cortex and receive dopaminergic input from the midbrain. The studies suggest that a circuit structure including VS, VTA/SN, ventral pallidum, prefrontal cortex and thalamus is an integral part of the cortico-basal ganglia system in reward circuit (Haber and Knutson, 2010). A meta-analysis of 142 neuroimaging studies of reward valence processing confirmed that different brain regions are broadly involved in different stages of reward processing such as the orbitofrontal cortex, anterior cingulate, a sub-region of the ventral striatum and the nucleus accumbens (Liu et al., 2011).

Functional MRI studies in humans have showed that different brain regions are engaged in reward expectation and reward processing. A common finding is that the ventral striatum, including the nucleus accumbens was preferentially activated during expectation of the reward, whereas the ventromedial prefrontal cortex preferentially activated during reward outcome (Knutson et al., 2001; O’Doherty et al., 2002). In another fMRI study focusing on decision-making outcome of the reward showed differential responses to reward and punishment in the dorsal and ventral striatum. Components of the dorsal striatum, showed an increase in activation that was more sustained for trials associated with a rewarding outcome than with a punishing outcome (Delgado et al., 2000).

While the above findings provide evidence for the view that reward prospect and time perception may act by utilizing partially overlapping processing routes, a systematic investigation of this proposed overlap, as well as the potential interaction between these two factors is lacking. With the present functional magnetic resonance imaging (fMRI) study, we sought to elucidate the neural processes that are shared and distinct between brain regions responsible for time perception and reward prospection.

## Materials and Methods

### Participants

Participants were 18 young adults (aged 18–45, mean = 25.8 ± 5.8 years, 11 female) with at least 8 years of school attendance. None of them reported a history of drug abuse, neurological or psychiatric diseases, or injuries to head. All were right handed according to the Chapman and Chapman Handedness Inventory (1987) (its validity and reliability for use in the Turkish population was reported by Nalçaci et al., 2002) with normal or corrected-to-normal visual acuity. The methods and procedures used in the study had approval from the Ankara University Institutional Review Board (AUIRB) and informed written consent was obtained from all participants in accordance with the protocols approved by the AUIRB. The participants were all volunteers and they have informed to receive a payment based on their performance prior to the study.

### Experimental Paradigm

To examine our hypothesis, we employed a temporal attention task in which observers had to extrapolate the velocity of an occluded moving object. The paradigm was designed using “Presentation^®^” (Version 18.0, Neurobehavioral Systems, Inc., Berkeley, CA, United States) program. The participants performed the tasks, while undergoing fMRI which consisted of two different conditions (control and time perception), applied in rewarded and unrewarded sessions.

On each trial, there was a black vertical bar in the middle of the screen with a gray background, which was constantly displayed during the trial. There was a unique cue associated with each condition and it was displayed in the center of the bar. For the time perception condition an arrow image was displayed as a cue and for the control condition a checkerboard was the cue.

After the presentation of the cue, the target which is a moving gray rectangle appeared from the left side of the screen and moved horizontally until it disappeared from the right side of the screen. When the rectangle reached the bar, part of it that was under the bar becoming “invisible” to the participant in order to induce the perception that the rectangle was passing under the bar. The initial speed of the rectangle was slightly increased or decreased while it was occluded by the bar but the rectangle resumed its initial speed when it reappeared on the right side of the bar. The color tone of rectangle gets lighter or darker from the initial level after the occlusion period.

In the time perception condition participants were asked to judge whether the rectangle reappeared earlier or later after the occlusion compared with its predicted velocity. They were required to press the right button of a response pad if the speed of the rectangle was increased while passing under the bar, and to press the left button if the speed of rectangle was decreased. In the control condition participants were asked to attend to the contrast change of rectangle when it was reappeared again after the occlusion. They were asked to evaluate whether the color tone of the rectangle was darker or lighter compared to its initial tone. They were required to press the right button in case the color tone was decreased and press left button if it was increased. The color tone and the velocity of the rectangle changed in both conditions. However, participants were asked to attend to either velocity or color contrast according to the cue represented in the middle of the bar. The participants were instructed to press the buttons as quickly as possible when they decided to answer but also reminded that giving correct answer is also important for both rewarded and unrewarded sessions. A fixation point was presented on a gray screen at the inter-stimulus intervals which were 2, 4, and 6 s arranged in a pseudo-randomized and logarithmic manner favoring shorter durations. The sessions were also presented in a pseudo-randomized order. Reaction times (RT) were collected and percentage of correct answers were calculated. In rewarded sessions, the participants gained money depending on their percentage of correct response (PCR) scores (100 Turkish Lira to 100% correct score).

The tasks were presented on a 28 cm × 37.5 cm screen with a distance of 72.5 cm from the participant’s eyes to the screen. The monitor resolution was 1920 pixels × 1080 pixels and the refresh rate was 60 Hz. The size of the black bar was 28cm × 9.47 cm (21.86° × 7.47°) and the size of moving rectangle was 1.46 cm × 1.94 cm (1.16° × 1.54°). The rectangle passed across the screen from left to right horizontally with two possible speeds when it is visible to the participant. The speeds of the rectangle were 435°/1817 ms and 435°/917 ms. If the initial speed of the rectangle was 3.70°/s it either increased to 7.11°/s or decreased to 1.82°/s while rectangle passed under the bar and when the rectangle reappeared again on the right side of the screen it returned to its initial speed. If the initial speed of rectangle was 7.32°/s it either increased to 10.48°/s or decreased to 3.62°/s while rectangle passed under the bar. Contrasts of the rectangle were calculated by using Weber contrast equation which is (pixel intensity of rectangle- pixel intensity of background)/pixel intensity of the background. The initial contrasts of the rectangle were -0.64, -0.52, and -0.37. If the initial contrast of rectangle was -0.64 it either increased to -0.76 or decreased to -0.52 when rectangle reappeared again on the right side of the screen. If the initial contrast of rectangle was -0.52 it either increased to -0.64 or decreased to -0.37 and if the initial contrast of rectangle was -0.37 it either increased to -0.52 or decreased to -0.21 when rectangle reappeared again on the right side of the screen. One trial lasted 2500 ms in total.

**FIGURE 1:**
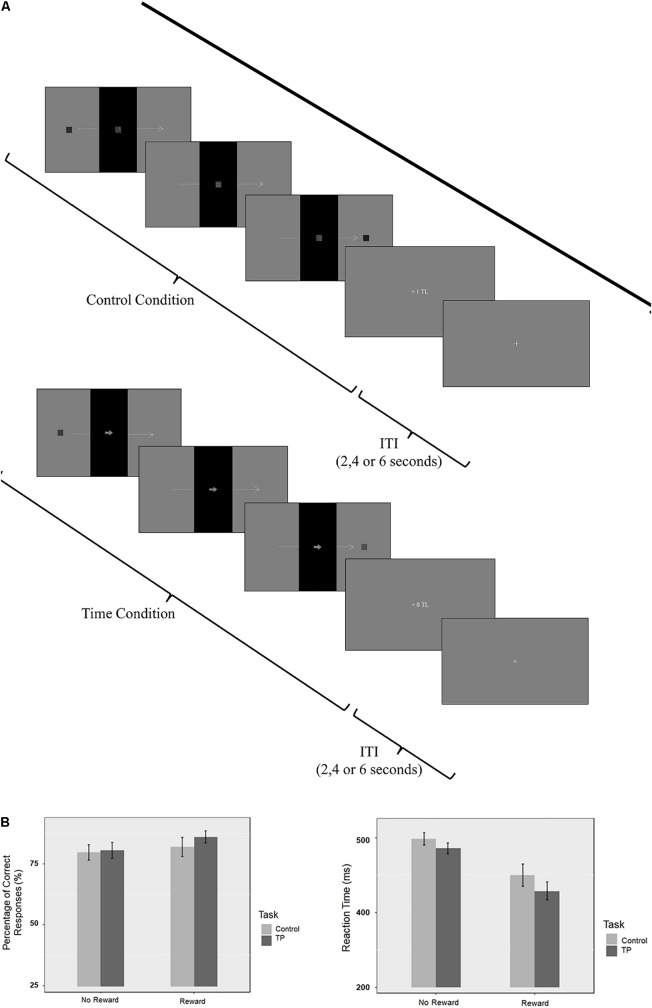
**(A)** Graphical representation of the sequence of events in each trial for reward conditions. **(B)** Percentage of correct responses (PCR) and reaction time (RT) results.

An event-related fMRI design was used. Before fMRI acquisition, all subjects performed a training session and had feedback if they had correctly done the trial or not. All participants succeed to finish the trial session with more than 60% accuracy. Inside the scanner, the participants performed four 7-min sessions, yielding a total of 32 trials in each session (16 trials for each condition). There were four sessions; two sessions were rewarded and two sessions were unrewarded (**Figure 1**).

### fMRI Image Acquisition

fMRI images were acquired using a 3-T Siemens Magnetom Trio MRI system with a 32-channel head-coil array. For each participant, a series of high resolution T1-weighted anatomical images were obtained [Time to repeat (TR): 2600, Time to Echo (TE):3.02, Field of View (FOV): 256 mm and slice thickness: 1.00 mm]. Functional scans were acquired using 46 3-mm slices with a 0-mm gap (TR:2600, TE:28, matrix:64 × 64, FOV: 192 mm, voxel size: 3 mm × 3 mm × 3 mm).

To display the visual stimuli Presentation^®^ was run via a PC which was also used for collecting participants’ response. The visual paradigm was projected onto a projection screen which was visible via a mirror that situated front of participant’s head. Participants used a response pad with their right hand to performed the tasks while undergoing fMRI scan.

### fMRI Processing and Data Analysis

Analysis of the fMRI data was performed using SPM8 software (Wellcome Department of Cognitive Neurology, London, United Kingdom) run via MATLAB. The functional images were realigned to correct for movement artifacts. High-resolution anatomical T1 images were coregistered with the realigned functional images to enable anatomical localization of the activations. The structural and functional images were spatially normalized into a standardized anatomical framework using the default EPI template in SPM8, based on the Montreal Neurological Institute (MNI) averaged brain and approximating the normalized probabilistic spatial reference frame of Talairach and Tournoux (1988). Model estimation included a high-pass filter (256s). Smoothing was performed with a 6-mm full-width half-maximum Gaussian kernel. The GLM design matrix included four task-related regressors (rewarded time, rewarded control, unrewarded time, unrewarded control). Also the six-head movement regressors derived from the realignment stage of preprocessing were also included as covariates of no interest.

The neuroimaging data were statistically analyzed by a 2 (time/no-time) × 2 (reward/no-reward) repeated measures analysis of variance (ANOVA) using the SPM12 flexible factorial design feature at the group level. The trials with incorrect subject responses were not included in the analysis. The averaged activity in the peak regions were extracted from individual subject images for the brain areas showing significant interaction solely to reveal the direction of the effect. To this end, mean percent signal change values were calculated for spheres centered at the peaks of activation clusters with 5 mm (for caudate nucleus only) or 10 mm diameters (please see **Table 1** for coordinates).

**Table 1:** Significant activations revealed by the 2 × 2 ANOVA (*p* < 0.05, corrected).

			MNI coordinates			
Cluster size	Brain region	Laterality	*X*	*Y*	*Z*	*Z*-score
**Main effect of time**						
2034	Inferior parietal lobe	R	58	-42	46	6.65
523	Inferior parietal lobe	L	-56	-48	46	4.76
3235	Dorsolateral/ventrolateral prefrontal cortex	R	48	36	-2	6.11
758	Ventrolateral prefrontal cortex	L	-48	46	-4	5.29
277	Intraparietal sulcus	R	32	-72	34	5.23
392	Middle temporal gyrus (MT/V5)	L	-56	-56	-4	5.12
489	Middle temporal gyrus (MT/V5)	R	62	-52	-4	4.81
676	Anterior cingulate cortex/supplementary motor area	Bilateral	4	32	44	4.88
277	Cerebellum	L	-12	-82	-30	4.57
**Main Effect of reward**						
1590	Peristriate cortex	L	-18	-88	-6	5.82
1689	Peristriate cortex	R	28	-88	8	4.85
373	Postcentral gyrus	Bilateral	-26	-46	70	4.39
154	Anterior insular cortex	R	-20	22	12	4.09
127	Posterior cerebellum	L	24	-68	-40	4.92
136	Anterior cerebellum	Bilateral	0	-42	-2	4.51
824	Nucleus accumbens	Bilateral	10	4	-2	3.85
	Thalamus	Bilateral	0	-6	6	4.50
	Caudate nucleus (head)	L	-4	12	-2	4.24
	Caudate nucleus (head)	R	6	16	0	3.77
	Caudate nucleus (tail)	L	-14	-12	20	3.61
	Caudate nucleus (tail)	R	12	-2	-14	3.97
**Interaction**						
230	Dorsolateral prefrontal cortex	L	-34	22	40	4.14
109	Dorsolateral prefrontal cortex	R	38	36	34	3.87
136	Orbitofrontal cortex	L	-28	54	2	3.70
146	Caudate nucleus	L	-16	36	4	4.40
	Medial prefrontal cortex	L	-18	46	-4	3.69

## Results

### Behavioral Results

The PCR and RT of all attendants were evaluated by repeated measures ANOVA using R software. The Bonferroni correction was applied for multiple comparisons. Separate two by two ANOVAs were applied to PCR and RT results with task (time perception/control) and reward (reward/no-reward) as factors (see **Figure 1** for behavioral results).

The main effect of Task was not significant [*F*(1,17) = 1.37; *p* = 0.257; ${\text{η}}_{\text{G}}^{2}$ = 0.009]. The main effect of Reward was also not significant [*F*(1,17) = 0.91; *p* = 0.353; ${\text{η}}_{\text{G}}^{2}$ = 0.021]. These findings suggest that there is no difference in terms of difficulty between task and control conditions. The interaction between the task and the reward condition was not significant [*F*(1,17) = 1.88; *p* = 0.188; ${\text{η}}_{\text{G}}^{2}$ = 0.004]. Even though the accuracy difference is not significant, there is still improvement in accuracy on the rewarded condition. PCR on the unrewarded control condition was 79.7%; unrewarded time perception condition was 80.6%; while the PCR on the rewarded control condition was 81.9% and rewarded time perception was 86.1%.

The repeated measures ANOVA for RT showed a significant main effect of Task [*F*(1,17) = 17,70; *p* < 0.001; ${\text{η}}_{\text{G}}^{2}$ = 0.033]. There was a significant main effect of Reward (*F* = 19.98; *p* < 0.0001; ${\text{η}}_{\text{G}}^{2}$ = 0.251). These findings showed that subjects responded slower during control condition compared to the time perception condition. Also findings suggest that subjects responded faster during rewarded sessions compared to the unrewarded sessions.

### Functional Imaging Results

#### The Main Effect of Time Perception

**FIGURE 2:**
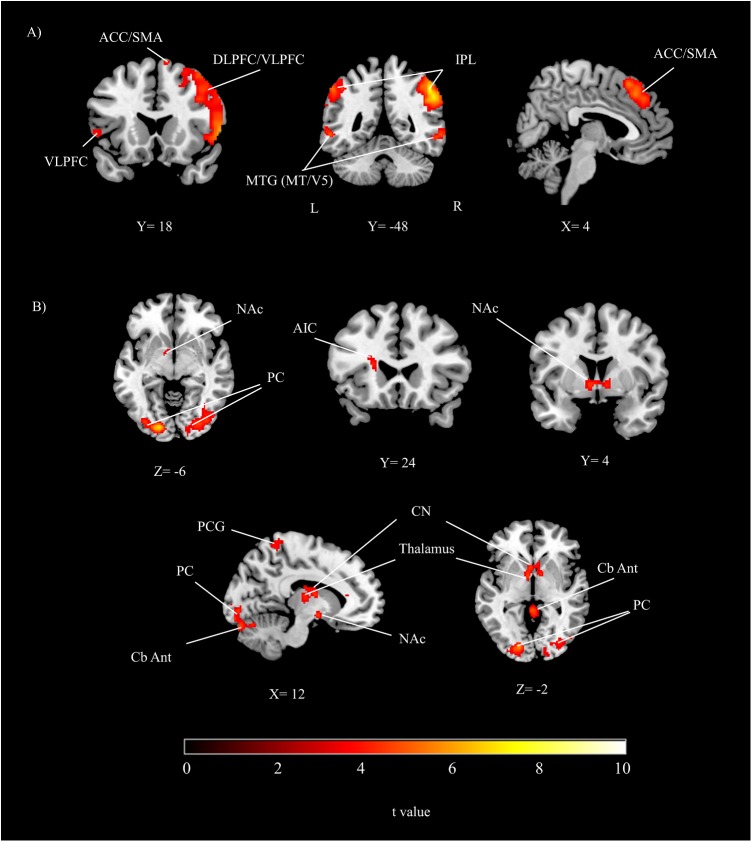
The group results depicting significant activations related to **(A)** the main effect of time perception, **(B)** the main effect of reward (threshold at *p* < 0.05, corrected). DLPFC, dorsolateral prefrontal cortex; VLPFC, ventrolateral prefrontal cortex; IPL, inferior parietal lobe; PC, peristriate cortex; ACC, anterior cingulate cortex; SMA, supplementary motor area; NAc, nucleus accumbens; AIC, anterior insular cortex; PCG, postcentral gyrus; CN, caudate nucleus; MTG, middle temporal gyrus; Cb Ant, cerebellum anterior; A, anterior; P, posterior; L, left; R, right.

The group results showed that while participants were performing the time perception task, inferior parietal lobe, dorsolateral/ventrolateral prefrontal cortex, intraparietal sulcus, peristriate cortex, ACC/SMA and cerebellum were significantly activated (**Table 1** and **Figure 2A**). Activity show a right hemisphere lateralization. Prefrontal and parietal cortex activity were more extensive in the right hemisphere. Overall, a distributed neural network was significantly activated during the timing task compared to the control condition.

#### The Main Effect of Reward

During the reward conditions, peristriate cortex, precuneus, anterior insular cortex (AIC) were significantly activated. In addition to the cortical activations posterior and anterior part of cerebellum, NAc, thalamus and caudate nucleus (CN) were significantly activated (**Table 1** and **Figure 2B**).

#### Interaction Between Time Perception and Reward

**FIGURE 3:**
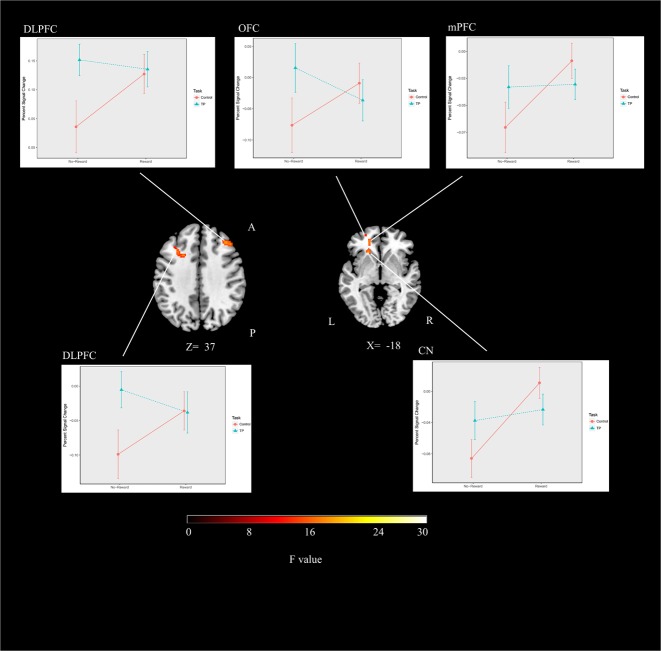
The brain activations and related graphs of the interaction of time perception and reward for the whole group (threshold at *p* < 0.05, corrected). DLPFC, dorsolateral prefrontal cortex; OFC, Orbitofrontal cortex; mPFC, medial prefrontal cortex; CN, caudate nucleus; A, anterior; P, posterior.

In this study, significant activations were found for the interaction between time perception and reward prospect. Activations were seen in bilateral dorsolateral prefrontal cortex, orbitofrontal cortex, medial prefrontal cortex and CN (**Table 1** and **Figure 3**).

ANOVA was performed for percent signal change values obtained from spheres at the peaks of significant interaction activation clusters. The results showed significant time and reward interaction effect for the right dorsolateral prefrontal cortex [*F*(1,17) = 11.23, *p* < 0.01], left dorsolateral prefrontal cortex [*F*(1,17) = 8.64, *p* < 0.01], orbitofrontal cortex [*F*(17) = 6.62, *p* < 0.05], medial prefrontal cortex [*F*(1,17) = 6.29, *p* < 0.05] and CN [*F*(1,17) = 12.15, *p* < 0.01] (**Figure 3**). The main effect of time was significant for left dorsolateral prefrontal cortex [*F*(1,17) = 5.04, *p* < 0.05] and right dorsolateral prefrontal cortex [*F*(1,17) = 16.11, *p* < 0.001]. The main effect of reward was significant for CN [*F*(1,17) = 13.67, *p* < 0.01].

Follow-up *t*-statistics analysis of percent signal change values obtained from ROIs showed that there was no difference in time perception condition between the rewarded and unrewarded sessions (*p* > 0.05). On the other hand, control condition showed more activity during the reward sessions compared to the unrewarded ones (*p* < 0.05). These results suggest that significant ANOVA effects were derived by brain activity difference observed for color contrast perception task related to reward anticipation but not for the time perception task.

## Discussion

We assessed common and distinct neural processes among time perception and reward prospection in healthy subjects by way of a visual paradigm that includes monetary reward. Time perception showed a fronto-parietal (more extensive in the right), ACC/SMA and peristriate cortical as well as cerebellar activity. On the other hand, reward anticipation activated AIC, NAc, CN, thalamus, cerebellum postcentral gyrus and peristriate cortex. The findings suggest that mainly a prefrontal subcortical circuit (prefrontal-caudate) may be responsible for the integration of time perception and reward prospect functions.

### Time Perception

Presented results showed that time perception activated bilateral prefrontal, IPL and IPS regions but more extensive (predominantly) in the right hemisphere. These findings almost replicated the results reported recently. Üstün et al. (2017) used a very similar foreperiod paradigm to research on the relationship of time perception and working memory. They showed right lateralized fronto-parietal activations related to timing. In fact, their time perception related specific activations obtained by time > memory contrast (Talairach Coordinates: 52, -37, 40) was approximately in the same location as in the presented studies’ time perception effect in parietal lobe (MNI Coordinates: 58, -42, 46). Prefrontal activity was also in close proximity in both studies. These findings support the view that while prefrontal cortex mediates the working memory aspect of timing, the IPL engages in the attentional mechanisms of time perception with a right hemisphere bias which might be the result of visuospatial nature of our foreperiod paradigm (Miall, 2006; Çiçek et al., 2009).

Bilateral SMA and ACC activations are also a replication of our lab’s previous results (Üstün et al., 2017) and also in line with other study’ findings (O’Reilly et al., 2008, 2013). It is suggested that SMA/ACC activity during timing might serve for internal model update and decision processes (Pouthas et al., 2005; O’Reilly et al., 2013).

The significant activity in V5/MT area is also in line with the previous neuroimaging studies using timing paradigms (Bueti et al., 2008b; Üstün et al., 2017). V5/MT area is suggested to be related to the perception of motion (Born and Bradley, 2005). Rather than just engaging related to the visual motion processing, V5/MT was suggested to be involved in visual perception of duration (Bueti et al., 2008a,b). The presented significant activity result in the cerebellum might also be related to the timing of moving objects (O’Reilly et al., 2008). Our paradigm required subjects to predict the timing of the reappearance of the occluded stimuli which might engage the cerebellar mechanisms.

### Reward Anticipation

We designed a visual paradigm using a secondary incentive (money) to engage reward anticipation processes. In the half of the scanning sessions subjects were awarded and in the other half they were not awarded (two sessions each). The main effect of reward, in another way of explanation, means result obtained by subtracting unrewarded from rewarded trials. The modeled activations are related to the reward anticipation rather than reward outcome. Also our paradigm do not include punishment, instead -during reward sessions- subjects are rewarded for correct answers and not rewarded for incorrect answers. Indeed, only the trials with correct subject responses were included in the analysis.

Key findings of the presented study for reward effect was the NAc, CN, and AIC activity which are proposed to be among the key structures of the reward circuit (Haber and Knutson, 2010). These findings are in line with the previous neuroimaging studies reporting the dorsal (CN) and ventral striatum (NAc) as well as AIC activity during reward anticipation (Breiter et al., 2001; Knutson et al., 2001, 2003; Zink et al., 2004; Knutson and Greer, 2008). A meta-analysis of cued response studies suggested that the NAc and AIC were activated during anticipation of uncertain incentives (Knutson and Greer, 2008). The same study also proposed that while NAc activity is related to the gain anticipation, AIC activates for both gain and loss anticipation.

### Integrating Time and Reward

Significant contribution of our study is the localization of the physiological brain mechanisms integrating time perception and reward prospect. Presented results suggest that a prefrontal-striatal circuit might be the hub for integrating time and reward networks. Interaction effect showed activity in the dorsolateral, orbitofrontal, medial prefrontal, and CN. Percent signal change values obtained from these brain areas suggest that these regions are activated during the rewarded but not during the non-rewarded control condition. This finding might be interpreted as these regions are affected by the reward manipulation. The activities in these regions are significantly greater during the non-rewarded timing condition compared to the non-rewarded control condition which suggests that timing engages these brain regions too. On the other hand, the rewarded timing condition activity was not greater than the non-rewarded timing condition (but was greater than the non-rewarded control condition). Overall these findings suggest that the prefrontal-striate neural network might be involved in both the timing and the reward processes.

The orbitofrontal, medial prefrontal cortex and CN were suggested to be activated during reward anticipation (Kim et al., 2006; Knutson and Greer, 2008). On the other hand, the medial prefrontal cortex is suggested to be activated specifically during the reward outcome (Knutson et al., 2003). Monetary reward related action selection was suggested to engage the orbital and dorsolateral prefrontal cortex activity for exploratory decisions, but the medial prefrontal for exploitative decisions (Daw et al., 2006). Dorsolateral prefrontal cortex is suggested to be related to choosing the most valuable among multiple options, which might require working memory (Haber and Knutson, 2010). On the other hand, CN activity was proposed to link reward to behavior (Knutson and Cooper, 2005).

The presented study showed ventral striatum activity during the rewarded sessions, but we also found dorsal caudate activity related to the reward processing. Presented work did not report dorsal striatum activity related to timing but caudate activity was previously showed during a very similar fore-period task (Üstün et al., 2017). Animal studies and human neuroimaging studies (including subjects with cocaine addiction) suggest that while ventral striatum neurons respond to reward processing, dorsal striatum activate during the timing and other cognitive processes like spatial learning (van der Meer et al., 2010; Coull et al., 2011; Tau et al., 2014). On the other hand, it is also reported that the reward processing and reward based temporal learning activates both ventral and/or dorsal striatum (Delgado et al., 2000; Bischoff-Grethe et al., 2015; Tomasi et al., 2015). It was proposed that differences in response requirements might explain the differential activation of ventral/dorsal striatum (Bischoff-Grethe et al., 2015).

The nature of our paradigm, which aims to engage both the timing and reward networks, is consistent with everyday life but neither differentiate the anticipation from outcome nor the exploration from exploitation. Overall, presented results suggest that the integration of timing and reward networks might occur by the significant contribution of a prefrontal-striatal circuit. This circuit most likely links processes related to the reward based decisions, the timing of the events and the timing of the behavioral response as well (Haber, 2016).

What might be the reason of the presented co-activation of the prefrontal-striatal network in terms of brain’s anatomy and cellular physiology? Based on diffusion tensor imaging fiber tracking results, CN was showed to connect with dorsolateral, orbitofrontal and medial prefrontal cortex (Lehéricy et al., 2004; Draganski et al., 2008). Dopamine neurons in the substantia nigra and VTA were reported to respond to the timing of rewards (Hollerman and Schultz, 1998; Schultz et al., 2000). Dopamine neurons send direct axonal connections to the striatum and to a wide area of the prefrontal cortex (Haber and Knutson, 2010). CN suggested to act as network hub to integrate functions of the medial, orbital and dorsolateral prefrontal cortex (Averbeck et al., 2014; Haber, 2016). These prefrontal-striatal connections are suggested to be parallel and segregated but show significant convergence as well (Haber, 2016). The same cortico-striatal connections are suggested to be glutamatergic and modulated by the dopaminergic input to the striatum (Gardoni and Bellone, 2015). Results suggest that the physiological mechanisms and neural networks involved in the reward prospect and time perception might converge on the dopaminergic cortico-striatal circuit (Meck and Benson, 2002; Coull et al., 2004; Haber and Knutson, 2010). Overall co-activation of the prefrontal-striatal network might reveal the integration of time perception and reward systems through direct and/or indirect contributions of dopaminergic and glutamatergic neurons.

Since the presented work submitted to a special issue of time and number, a brief discussion of our findings in terms of number sense is required. It is proposed that the intraparietal sulcus and the prefrontal cortex of the primate are both involved in the encoding of space, time and number (Burr et al., 2010). Tasks requiring number and time magnitude estimations suggested engaging basal ganglia, prefrontal and parietal cortex activity (Allman et al., 2011). COMT-related dopaminergic mechanisms were suggested to be implicated in number processing (Julio-Costa et al., 2013). Findings suggest that while COMT polymorphism causing higher prefrontal dopaminergic activity affected supra-second timing performance, DRD2 polymorphism resulting in higher striatal D2 activity affected sub-second timing performance (Wiener et al., 2011). Balcı and his coworkers studied the effect of COMT and DRD2 polymorphism on the relation between time perception and reward anticipation. They found that gene polymorphism status related to balanced prefrontal (D1 receptor) and striatal (D2 receptor) activity, associated with the significant interaction of reward magnitude with timing performance (Balcı et al., 2013). Overall, the number processing, time perception and reward anticipation might engage substantially overlapping brain network probably depending on a cortico-striatal dopaminergic neural circuit (Wiener et al., 2011; Balcı et al., 2013; Julio-Costa et al., 2013).

## Conclusion

Our findings suggest that a prefrontal-striatal circuit might integrate reward and timing systems of the brain. Fundamental functions of the brain like spatial attention, time perception, working memory and as well as number sense were suggested to engage a fronto-parietal network (Burr and Morrone, 2006; Çiçek et al., 2009; Üstün et al., 2017). The prefrontal-striatal circuit might also be an important hub for integrating the reward system with other fronto-parietal network related systems of the brain.

## Author Contributions

NA and SÜ: substantial contributions to the conception and design of the work, acquisition, analysis, and interpretation of data and writing the work. EK: substantial contributions to the analysis and interpretation of data and revising the work critically. İÇ: substantial contributions to the acquisition of data. HÖ and BB: substantial contributions to the conception and design of the work and revising the work critically. MÇ: project supervision, substantial contributions to the conception and design of the work, analysis and interpretation of the data, writing and revising the work critically. NA, SÜ, EK, İÇ, HÖ, BB, and MÇ: final approval of the version to be published and agreement to be accountable for all aspects of the work.

## Conflict of Interest Statement

The authors declare that the research was conducted in the absence of any commercial or financial relationships that could be construed as a potential conflict of interest.
